# A plasma telomeric cell-free DNA level in unaffected women with BRCA1 or/and BRCA2 mutations: a pilot study

**DOI:** 10.18632/oncotarget.23767

**Published:** 2017-12-29

**Authors:** Shatovisha Dey, Natascia Marino, Kanokwan Bishop, Paige N. Dahlgren, Aditi Shendre, Anna Maria Storniolo, Chunyan He, Hiromi Tanaka

**Affiliations:** ^1^ Department of Medical and Molecular Genetics, Indiana University School of Medicine, Indianapolis, IN, USA; ^2^ Department of Medicine, Indiana University School of Medicine, Indianapolis, IN, USA; ^3^ Susan G. Komen Tissue Bank at IU Simon Cancer Center, Indianapolis, IN, USA; ^4^ Department of Epidemiology, Richard M. Fairbanks School of Public Health, Indiana University, Indianapolis, IN, USA; ^5^ Department of Internal Medicine, College of Medicine, Markey Cancer Center, University of Kentucky, Lexington, KY, USA

**Keywords:** BRCA1, BRCA2, telomere, circulating cell-free DNA, qPCR

## Abstract

Plasma cell-free DNA (cfDNA) is a small DNA fragment circulating in the bloodstream originating from both non-tumor- and tumor-derived cells. A previous study showed that a plasma telomeric cfDNA level decreases in sporadic breast cancer patients compared to controls. Tumor suppressor gene products including BRCA1 and BRCA2 (BRCA1&2) play an important role in telomere maintenance. In this study, we hypothesized that the plasma telomeric cfDNA level is associated with the mutation status of BRCA1&2 genes. To test this hypothesis, we performed plasma telomeric cfDNA quantitative PCR (qPCR)-based assays to compare 28 women carriers of the BRCA1&2 mutation with age-matched controls of 28 healthy women. The results showed that the plasma telomeric cfDNA level was lower in unaffected BRCA1&2 mutation carriers than in age-matched controls from non-obese women (BMI < 30), while there was no association between unaffected BRCA1&2 mutation carriers and age-matched controls in obese women (BMI > 30). Moreover, the plasma telomeric cfDNA level applied aptly to the Tyrer-Cuzick model in non-obese women. These findings suggest that circulating cfDNA may detect dysfunctional telomeres derived from cells with BRCA1&2 mutations and, therefore, its level is associated with breast cancer susceptibility. This pilot study warrants further investigation to elucidate the implication of plasma telomeric cfDNA levels in relation to cancer and obesity.

## INTRODUCTION

Plasma, serum, and other biofluids contain very low amounts of circulating extracellular cell-free DNA, also called cfDNA. cfDNA has become an increasingly important source for the development of liquid biopsy assays for early cancer detection. Indeed, it has been shown that cancer patients present both normal tissue- and tumor-derived cfDNA in the bloodstream [1]. Current cfDNA assays have mostly focused on monitoring cancer-specific mutations or methylation changes by targeting cell-free tumor DNA (ctDNA) [2]. In this case, the assays require prior information on specific genetic or epigenetic alterations present in the original tumor lesion. In order to detect cfDNA alterations associated with breast cancer initiation (where the amount of affected DNA is significantly low), the assays require sufficient improvement in the sensitivity of the assay, making this research area quite challenging [3, 4].

We recently devised a qPCR-based cfDNA assay to measure telomeric cfDNA levels (i.e., relative amounts of [TTAGGG]n sequences) in plasma. Using this assay, we reported that plasma telomeric cfDNA levels were significantly decreased in sporadic breast cancer patients with no prior treatment, compared to non-cancer controls [5]. While a plasma centromeric cfDNA level was also measured in the study and the difference between breast cancer patients and controls was also statistically significant, the actual effect size was modest between breast cancer patients and controls [5]. These results indicate that changes in telomeric cfDNA levels could be more constructive as a biomarker than changes in centromeric cfDNA levels during breast carcinogenesis. Furthermore, we found that the plasma telomeric cfDNA level in women with advanced breast tumors (stages II–III) was lower than those at earlier stages of breast cancer (stages 0–I) [5]. These findings suggest that telomere dysfunction (e.g., rapid telomere shortening) in body tissues might accumulate during cancer development and be associated with cancer progression, resulting in dynamic changes in the plasma telomeric cfDNA level.

Two major factors responsible for telomere length shortening are cellular replicative aging and genetics. BRCA1 and BRCA2 (BRCA1&2) are multifunctional proteins and maintain genomic stability by regulating DNA repair, transcription, and cell cycle in response to DNA damage [6, 7]. Several studies suggest that BRCA1&2 haploinsufficiency could involve defects in telomere maintenance [8–10]. Because *BRCA1&2* gene mutation carriers have a high risk of developing early-onset breast and ovarian cancer during their lifetime, the aim of this study is to determine whether plasma telomeric cfDNA level is associated with the BRCA1&2 mutations. This pilot study provides important supportive evidence for ensuring further research to determine the potential value of the plasma telomeric cfDNA level in predicting cancer development and cancer risk.

## RESULTS

**Figure 1:**
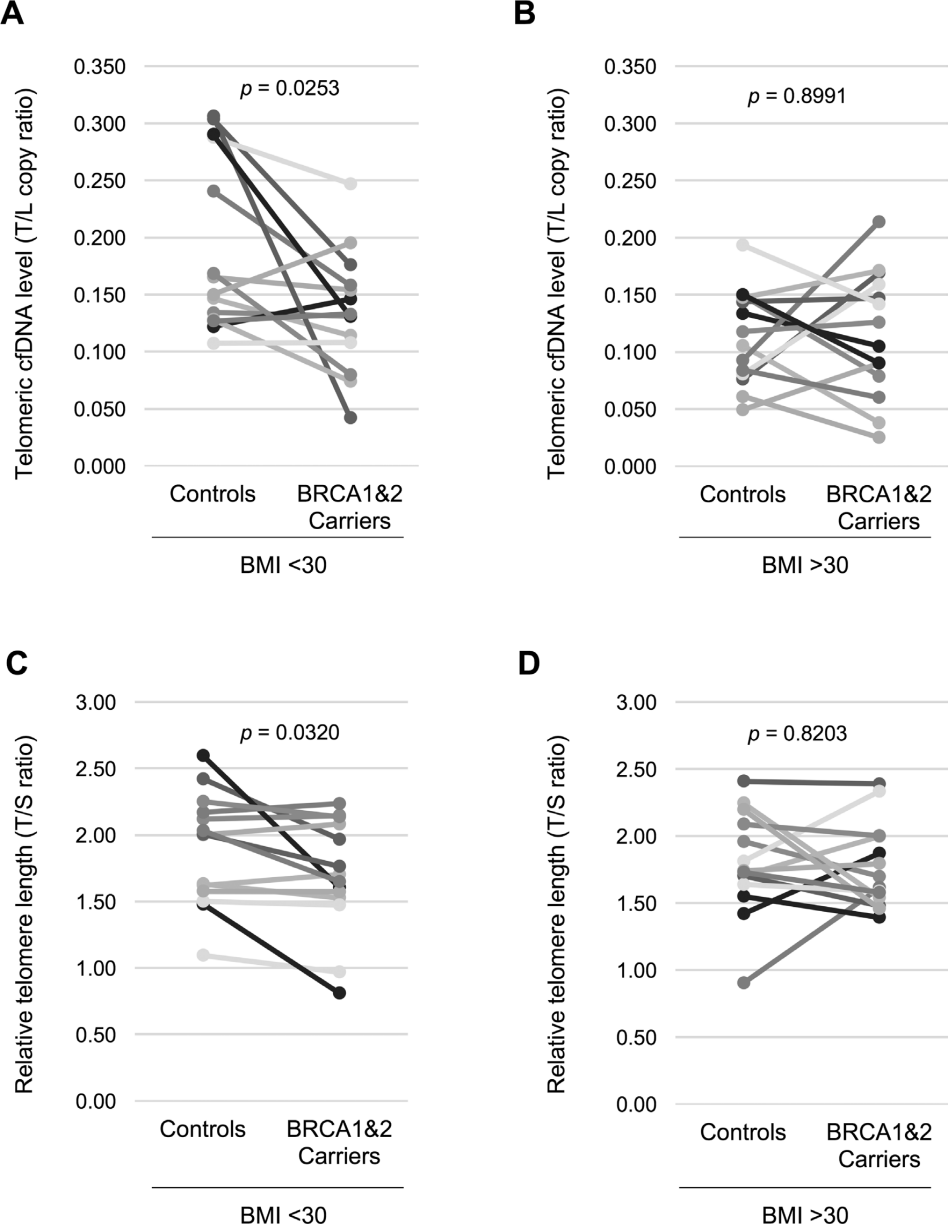
Non-obese women with BRCA1&2 gene mutation have a lower plasma telomeric cfDNA level and shorter leukocyte telomere length (**A**) The telomeric cfDNA level was plotted using 14 age-matched pairs from non-obese women (BMI < 30). Mean (± S.D.) of the telomeric cfDNA level was 0.191 (± 0.073) in controls and 0.135 (± 0.050) in BRCA1&2 carriers. 71.4% (10 of 14 pairs) show a reduction in the telomeric cfDNA level in BRCA1&2 carries. (**B**) The telomeric cfDNA level was plotted using 14 age-matched pairs from obese women (BMI > 30). Mean (± S.D.) of the telomeric cfDNA level was 0.115 (± 0.040) in controls and 0.113 (± 0.053) in BRCA1&2 carriers. 50.0% (7 of 14 pairs) show a reduction in the telomeric cfDNA level in BRCA1&2 carries. (**C**) Relative telomere length was plotted using the same pairs from non-obese in (A). Mean (± S.D.) of telomeric cfDNA level was 1.85 (± 0.45) in controls and 1.69 (± 0.41) in BRCA1&2 carriers. 71.4% (10 of 14 pairs) show a reduction in the telomeric cfDNA level in BRCA1&2 carries. (**D**) Relative telomere length was plotted using the same pairs from obese women in (B). Mean (± S.D.) of telomeric cfDNA level was 1.79 (± 0.37) in controls and 1.77 (± 0.30) in BRCA1&2 carriers. 64.2% (9 of 14 pairs) show a reduction in the telomeric cfDNA level in BRCA1&2 carries. All *p* values were shown based on paired Student’s *t* test.

We hypothesized that a circulating telomeric cfDNA level could be affected by BRCA1&2 heterozygous mutations; consequently, the telomeric cfDNA level decreases in BRCA1&2 mutation carriers as compared to healthy controls. In order to avoid reverse causation, we tested this hypothesis only in unaffected women. We analyzed plasma samples from 56 individuals, including 28 unaffected women carrying either a BRCA1 mutation (*n* = 16), a BRCA2 mutation (*n* = 9), or both BRCA1 and BRCA2 mutations (*n* = 3), and 28 age-matched healthy controls. The age range was from 23 to 74. Demographic and clinical characteristics of both cases and controls are provided in Table 1 and Supplementary Table 1, respectively. Telomeric cfDNA qPCR assay was performed as previously described with minor modifications [5]. Both parametric and non-parametric tests were employed to compare the telomeric cfDNA qPCR results collected from the two groups (carriers and controls). We first used paired *t*-test to compare the results between age-matched BRCA1&2 carriers vs. control women, thereby eliminating the confounding factor of age. Overall, there was no difference of the telomeric cfDNA level between BRCA1&2 carriers and controls (*p* = 0.0774, *d* = 0.351). However, upon subgrouping the samples by BMI, the plasma telomeric cfDNA level was significantly lower in BRCA1&2 carriers than those in age-matched controls in the non-obese group (*n* = 14 pairs, *p* = 0.0253, *d* = 0.691) (Figure 1A). In contrast, there was no difference in the telomeric cfDNA level between BRCA1&2 carriers and age-matched controls in the obese group (*n* = 14 pairs, *p* = 0.8991, *d* = 0.036) (Figure 1B). Wilcoxon signed-rank test showed that the *p*-value was also significant when BRCA1&2 carriers were compared with age-matched controls in the non-obese group (*p* = 0.0257), while the *p*-value was 0.976 in the obese group (Figure 1B). The effect size (difference) between BRCA1&2 carriers and age-matched controls in the non-obese group was a range from medium to large (*d* = 0.691). We also measured relative telomere length by qPCR in matched peripheral blood DNA samples as several reports have shown BRCA1&2 carriers having relatively shorter telomeres in leukocytes as compared to healthy controls [10, 11]. Our analyses using paired *t*-test showed that leukocyte telomere length was significantly shorter in BRCA1&2 carriers than those in age-matched controls in the non-obese group (*p* = 0.0320, *d* = 0.666) but not in the obese group (*p* = 0.8203, *d* = 0.064) (Figure 1C and 1D). However, there was no association between the plasma telomeric cfDNA level (T/L copy ratio) and leukocyte telomere length (T/S ratio) in this study population (Supplementary Figure 1), supporting our previous findings that leukocyte DNA is not a major source of cfDNA production [5].

**Table 1:** Demographic and clinical characteristics, telomeric cfDNA level, and telomere length of BRCA1&2 carrier women

Sample ID	Age	Race	BMI	Current smoking status	Menstrual Status	Mutation Status	10-year risk	Telomeric cfDNA level (± S.E.)	Relative telomere length (± S.E.)
BR02-469	23	White	27.3	No	Pre	BRCA1	4.9%	0.176 (± 0.0039)	1.97 (± 0.080)
BR27-689	23	White	32.1	No	Pre	BRCA2	5.7%	0.169 (± 0.0044)	2.39 (± 0.093)
BR12-497	24	White	28.5	No	Pre	BRCA1	8.6%	0.114 (± 0.0071)	1.70 (± 0.104)
BR31-071	24	White	24.3	No	Pre	BRCA1	19.0%	0.131 (±0.0009)	2.14 (± 0.084)
BR03-560	29	White	27.3	No	Pre	BRCA1	26.3%	0.146 (± 0.0079)	1.60 (± 0.095)
BR05-977	32	White	26.2	No	Pre	BRCA2	19.4%	0.247 (± 0.0621)	0.97 (± 0.054)
BR24-030	34	White	32.0	No	Pre	BRCA1	66.7%	0.038 (±0.0055)	2.00 (± 0.167)
BR07-177	37	White	33.9	No	Post	BRCA1 & BRCA2	18.8%	0.079 (± 0.0064)	1.70 (± 0.049)
BR34-032	37	White	21.1	No	Pre	BRCA2	16.1%	0.154 (± 0.0067)	2.08 (± 0.151)
BR11-468	38	White	27.3	Yes	Pre	BRCA1	23.8%	0.158 (± 0.0077)	2.24 (± 0.130)
BR14-590	39	White	32.8	No	Pre	BRCA2	18.8%	0.105 (± 0.0091)	1.87 (± 0.136)
BR35-034	41	White	31.6	No	pre	BRCA1	30.1%	0.159 (± 0.0079)	2.33 (± 0.070)
BR06-978	42	White	19.1	No	Post	BRCA2	17.2%	0.042 (± 0.0050)	1.76 (± 0.032)
BR13-588	45	White	31.2	No	Post	BRCA2	22.8%	0.090 (± 0.0071)	1.79 (± 0.067)
BR26-158	45	White	39.4	No	n/a	BRCA1	33.7%	0.214 (± 0.0175)	1.61 (± 0.093)
BR01-938	46	White	22.7	No	Post	BRCA1	32.8%	0.074 (± 0.0048)	1.76 (± 0.110)
BR28-925	46	White	32.1	No	Pre	BRCA2	21.5%	0.147 (± 0.0046)	1.48 (± 0.059)
BR16-766	47	White	35.7	No	Post	BRCA1	34.9%	0.171 (± 0.0185)	1.54 (± 0.110)
BR20-204	48	White	25.8	No	Post	BRCA1	19.9%	0.080 (± 0.0032)	2.14 (± 0.121)
BR32-664	49	White	32.6	No	Post	BRCA1	28.4%	0.126 (± 0.0026)	2.00 (± 0.068)
BR04-976	50	White	26.6	No	Pre	BRCA2	22.8%	0.131 (± 0.0074)	0.81 (± 0.022)
BR30-175	51	White	35.4	No	Post	BRCA1	26.3%	0.090 (± 0.0029)	1.39 (± 0.036)
BR29-168	53	White	32.2	No	Post	BRCA1	26.9%	0.142 (± 0.0086)	1.59 (± 0.039)
BR36-383	56	White	29.3	No	Post	BRCA1 & BRCA2	28.2%	0.108 (± 0.0063)	1.47 (± 0.111)
BR19-102	61	White	37.3	No	Post	BRCA1 & BRCA2	27.9%	0.025 (± 0.0014)	1.46 (± 0.061)
BR33-750	66	White	27.1	No	Post	BRCA1	12.6%	0.195 (± 0.0054)	1.57 (± 0.152)
BR21-207	70	White	22.6	No	Post	BRCA2	29.5%	0.133 (± 0.0163)	1.65 (± 0.142)
BR17-822	74	White	33.5	No	Post	BRCA1	8.5%	0.060 (± 0.0071)	1.58 (± 0.065)

^*^S.E., standard error of the mean.

**Figure 2:**
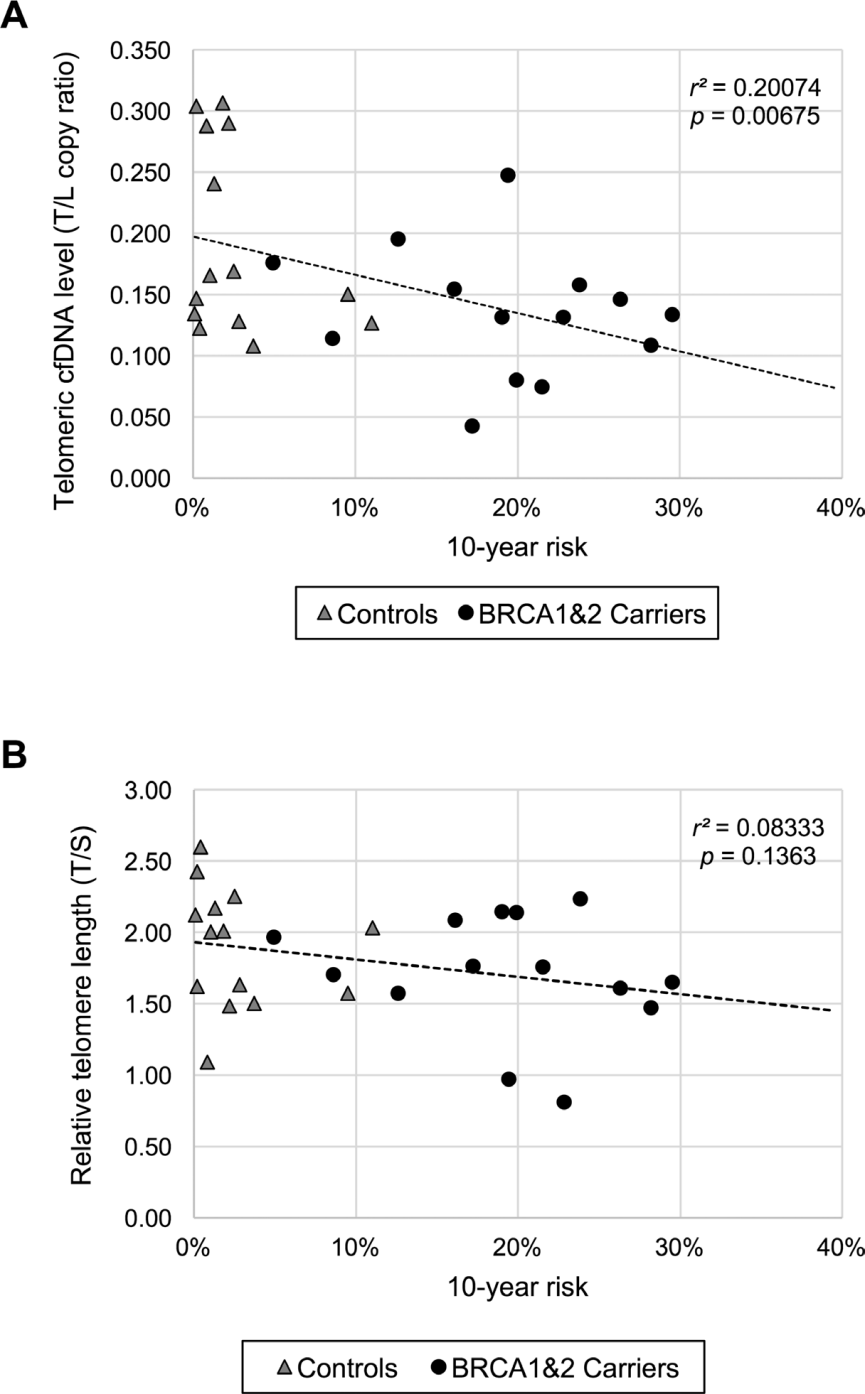
The plasma telomeric cfDNA level is correlated with individual 10-year breast cancer risk in non-obese women (**A**) Comparison between the plasma telomeric cfDNA level and the 10-year risk in non-obese women (*n* = 28). (**B**) Comparison between relative leukocyte telomere length and the 10-year risk in non-obese women (*n* = 28).

To further evaluate our results with other method, we used the Tyrer-Cuzick model that is a well-studied, widely available model for predicting breast cancer risk [12, 13]. We hypothesized that either telomeric cfDNA level or telomere length or both fit with the Tyrer-Cuzick model in non-obese women, thus acting as potential biomarker(s) for breast cancer development. When individual risk of developing breast cancer within 10 years was calculated and the plasma telomeric cfDNA level was compared with the 10-year risk, we found that the telomeric cfDNA level was inversely correlated with the 10-year risk (*p* = 0.00675, *r*^*2*^ = 0.2007), while the telomere length results were not significantly correlated with the 10-year risk (*p* = 0.1363, *r*^*2*^ = 0.0083) (Figure 2). Only when the data was analyzed in the non-obese controls (the 10-year risk < 5%, *n* = 22), the telomere length results were correlated with the 10-year risk (*p* = 0.0161, *r*^*2*^ = 0.2565). This significant correlation is likely attributed to age because the age factor is strongly associated with the 10-year risk score especially when the risk is less than 5% (Supplementary Figure 2).

**Figure 3:**
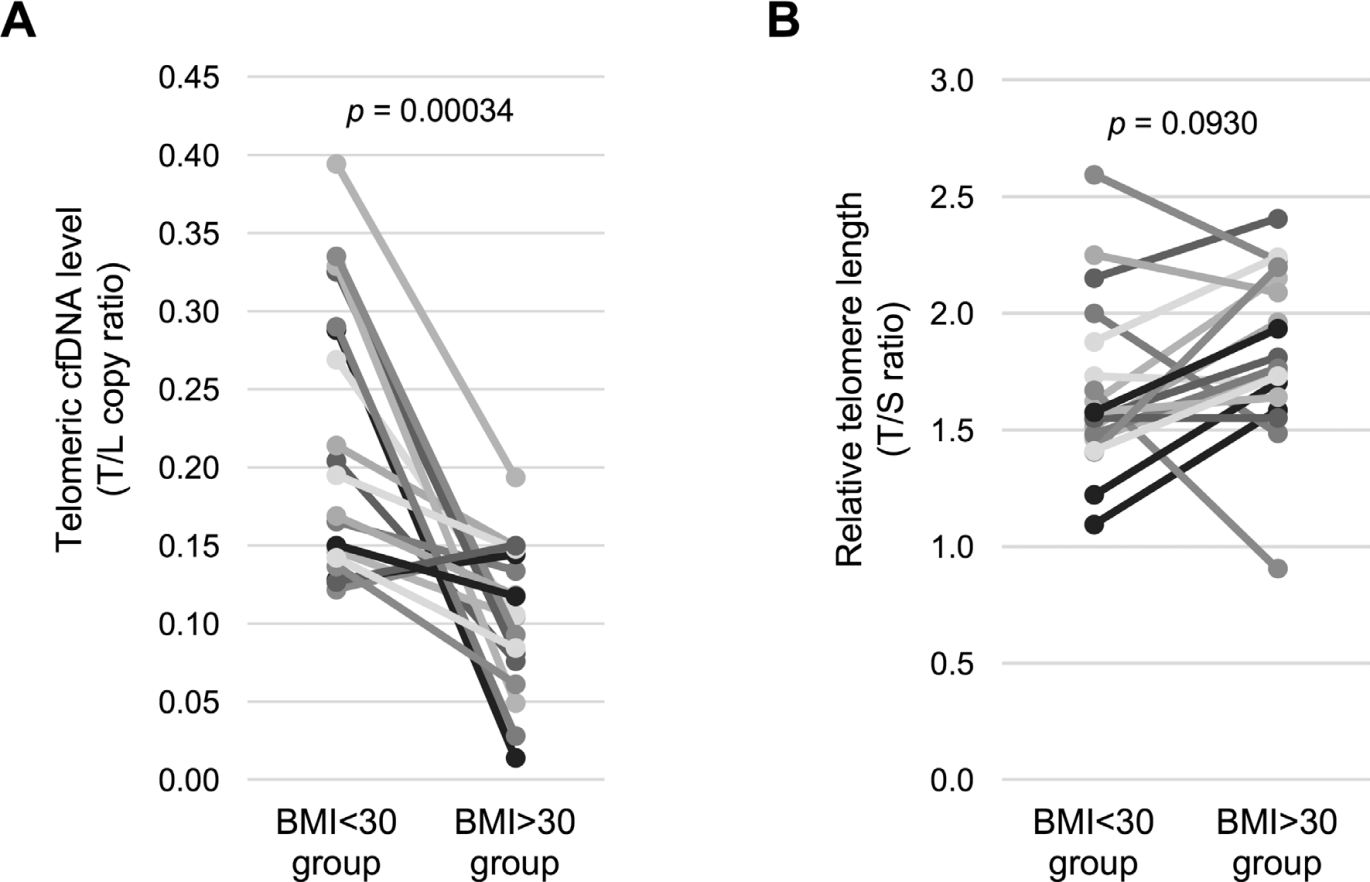
Healthy obese women have a lower telomeric cfDNA level than healthy non-obese women (**A**) The telomeric cfDNA level was sub-grouped by BMI using age-matched control women (*n* = 19 pairs). Mean (± S.D.) of the telomeric cfDNA level was 0.217 (± 0.084) in the BMI < 30 group and 0.105 (± 0.046) in the BMI > 30 group. 84.2% (16 of 19 pairs) show a reduction in the telomeric cfDNA level in the BMI > 30 group. (**B**) Relative telomere length was sub-grouped by BMI using age-matched control women (*n* = 19 pairs). Mean (± S.D.) of telomere length was 1.67 (± 0.35) in the BMI < 30 group and 1.83 (± 0.34) in the BMI > 30 group. There is no difference of telomere length between two groups. All *p* values were shown based on Wilcoxon signed-rank test.

Our findings indicate that obesity might have an effect on plasma telomere cfDNA level or/and leukocyte telomere length. Therefore, we next compared the difference in the plasma telomeric cfDNA level among 19 age-matched low (< 30) and high (> 30) BMI healthy control pairs. Non-parametric Wilcoxon signed-rank test was employed and showed that a lower plasma telomeric cfDNA level was associated with obesity (BMI > 30) in healthy individuals (*p* = 0.00034, *d* = 1.0667) (Figure 3A, Supplementary Table 2). In contrast, we did not find the difference in leukocyte telomere length between the obese and the non-obese groups (*p* = 0.093, *d* = 0.421) (Figure 3B, Supplementary Table 2), suggesting that obesity (based on BMI) is not a strong modifier of telomere length [14, 15].

## DISCUSSION

This pilot study is the first to determine the association of the plasma telomeric cfDNA level with genetic risk in unaffected women with and without mutations in *BRCA1* or/and *BRCA2* genes. The findings suggest that circulating plasma telomeric cfDNA levels are affected by BRCA1&2 heterozygous mutations in non-obese women. Until now, conflicting studies report the correlation between shorter telomere length in leukocyte-derived DNA and BRCA1&2 mutation status. Our results confirmed that leukocyte telomere length was affected by BRCA1&2 heterozygous mutations in non-obese women (but not in obese women). Furthermore, it is noteworthy that there is a significant correlation between the telomeric cfDNA level and the Tyrer-Cuzick 10-year risk score of developing breast cancer. These findings support our hypothesis that the telomeric cfDNA level could be a biologically-relevant quantitative biomarker for breast cancer development.

Although the underlying molecular mechanism of alteration in the plasma telomeric cfDNA level remains unclear, several studies reported that epithelial breast cells possess enhanced proliferative potential in women with BRCA1&2 haploinsufficiency [16–18]. We speculate that after the occurrence of “one-hit” of *BRCA1&2* gene, the remaining wild-type allele of *BRCA1&2* may not be sufficient to recover the deficiency in DNA repair and/or replication properly at the telomeres in target somatic tissues. Consequently, a telomere shortening rate in cells of BRCA1&2 carriers could be accelerated during development and aging, compared to controls [19]. Thus, we speculate that the circulating telomeric cfDNA level could reflect the somatic and dynamic changes in telomere dysfunction in target tissue cells; as a result, we observed that BRCA1&2 carriers have relatively lower telomeric cfDNA level than controls, even before a total loss of the remaining wild-type allele occurs and cancer appears. Moreover, we demonstrated that a lower plasma telomeric cfDNA level was associated with a higher BMI. The question of why the plasma telomeric cfDNA level is lower in obese women requires further investigation; however, we pose the following two possible explanations. First, adipocyte cells may have relatively shorter telomeres [20], and therefore increased death of the adipocyte cells results in a lower telomeric cfDNA level in plasma. Alternatively, obesity-related metabolic changes may induce higher enzymatic activity (e.g., nuclease) in the bloodstream that in turn facilitate degradation of cfDNA fragments outside-in [21]. Therefore, it is of value to further studying the implications of plasma telomeric cfDNA levels in cancer development and obesity.

Because we only included Caucasian women, these findings may not be generalized to men and/or other ethnicities. In addition, because the sample size in this study was limited, we were not able to identify differences in the plasma telomeric cfDNA level between BRCA1 carriers, BRCA2 carriers, and dual carriers. This study also lacks information about the specific mutation site(s) on *BRCA1&2* genes. Investigating the correlation of the changes in the plasma telomeric cfDNA level with specific BRCA1&2 mutation sites may provide new insights into risk assessments of breast cancer.

In summary, we found that *BRCA1&2* heterozygous mutations altered the plasma telomeric cfDNA level in non-obese women. This work highlights that the plasma telomeric cfDNA level may reflect the dynamic changes occurring in the telomeres of the somatic tissue cells as a result of genetic predisposition (i.e., BRCA1&2 mutations). The next steps would be to replicate the findings in a larger study. It is also critical to examine the prospective associations between the plasma telomeric cfDNA level, predictive risk factors for breast cancer, and breast cancer occurrence, and also to further determine whether the plasma telomeric cfDNA level mediates the association between these risk factors and breast cancer.

## MATERIALS AND METHODS

### Human specimens

Paired leukocyte DNA and frozen plasma samples were collected from BRCA1&2 mutation carriers (*n* = 28, 23 ≤ age ≤ 74, mean age = 46) and their age-matched controls (*n* = 28, ± 1 year) by the Susan G. Komen Tissue Bank at Indiana University Simon Cancer Center (KTB) with an approved Indiana University Institutional Review Board protocol. All samples were collected in accordance with standard operating procedures described on the KTB website and with donor’s written informed consent. All BRCA1&2 carrier cases and healthy controls were unaffected women. Of note, the BRCA1&2 mutation status of controls was based on self-reported information. The demographical and clinical information of the study subjects were collected by the KTB through a baseline questionnaire, and were shown in Table 1 and Supplementary Table 1. Body Mass Index (BMI) was calculated as weight divided by squared height.

### Plasma cfDNA extraction

Frozen plasma samples were thawed on ice prior to cfDNA isolation and centrifuged for 3 min at ≥ 11,000 × *g* in order to remove residual cells, cell debris, and particulate matter. A sodium Iodide (NaI)-based method was used for each cfDNA extraction as described previously [5]. cfDNA concentrations were measured using Quant-iT™ PicoGreen™ dsDNA Reagent (Thermo Fisher Scientific). The fluorescence intensity was measured with a Synergy 2 Multi-Mode Reader at emission wavelength of 520 nm and excitation wavelength of 480 nm.

### Telomeric cfDNA qPCR assay

Telomeric cfDNA levels were measured by qPCR as described previously except for some minor modifications [5]. The qPCR was performed on QuantStudio 6 Flex Real-Time PCR System (Thermo Fisher Scientific) in 10 μL volume of PCR reaction with 1× SYBR Select (Thermo Fisher Scientific), primers, and cfDNA (∼50 pg) as template. The primer sequences are shown in Supplementary Table 3. The thermal cycling profile is Stage 1 for 2 min at 95°C; Stage 2 for 2 cycles of 15 s at 94°C, 15 s at 49°C; and Stage 3 for 40 cycles of 15 s at 94°C, 15 s at 60°C, 20 s at 72°C. The qPCR results were analyzed based on plasmid DNA standard curves. The plasmid DNA contains (TTAGGG)_13_ and 121 bp of LINE (Long INterspersed Element) nucleotide sequences. Five concentrations of a plasmid DNA sample were prepared by five serial dilutions (3.62 × 10^9^ to 5.79 × 10^6^ copies). To monitor and compensate for inter-plate variations in PCR efficiency, each plate included standard plasmid DNAs as well as reference DNA (Promega, G152A). All experimental DNA samples were repeated at least three times in duplicate. All samples have a standard deviation of less than 0.5 for the threshold cycle (Ct) values. The inter- and intra-assay coefficients of variations (CVs) were 13.0% and 3.9%, respectively. Melting curve analysis was performed on every run to verify specificity and identity of the PCR products. The telomeric cfDNA levels were presented as T/L copy ratios (= telomere copy number/Line copy number) calculated from the average of more than three independent experiments.

### Leukocyte DNA extraction

Paired leukocyte DNAs along with plasma from both case and control women were obtained as a lyophilized power after the Komen Tissue Bank was extracted from whole blood samples using the Flex Star automated system with the AGFStar Fresh WB Extraction Kit (Autogen). The DNA was rehydrated with UltraPure DNase/RNase-free water (Thermo Fisher Scientific, 10977015). The DNA concentration was quantitated by Nanodrop 2000 spectrophotometer (Thermo Fisher Scientific).

### Relative telomere length measurement

Singleplex telomere length qPCR was performed with a QuantStudio 6 Flex Real-Time PCR System and analyzed as described previously [5, 22, 23]. The qPCR was performed on QuantStudio 6 Flex Real-Time PCR System (Thermo Fisher Scientific) in 10 μL volume of PCR reaction with 1× SYBR Select (Thermo Fisher Scientific), primers, and DNA as template. The telomere primers (telg and telc) and β-globin gene primers (hbgu and hbgd) were used for this assay. Each DNA standard curve was generated using a pooled female genomic DNA (Promega, G152A) and used for calculation of the T/S ratios (= ratios of “telomere quantities/single copy gene quantities”). All experimental DNA samples were repeated at least three times in duplicate. All replicate samples had a standard deviation of less than 0.5 for the threshold cycle (Ct) values.

### Breast cancer risk assessment

The Tyrer-Cuzick (or International Breast Intervention Study, IBIS) model was used to estimate a woman’s risk of developing breast cancer within the next 10 years using information about BRCA1/2 mutation carrier status and other risk factors including family history of breast and ovarian cancer, age at menarche, parity, age at first childbirth, age at menopause, atypical hyperplasia, lobular carcinoma *in situ*, height, and body mass index. The risk factors were entered into the model using the freely available software IBIS_RiskEvaluator_v8b (www.ems-trials.org/riskevaluator) [12, 13].

### Statistical analysis

Shapiro-Wilk test was used to test for normality in each group. Student’s *t* test (parametric) and Wilcoxon signed-rank test (non-parametric) were performed to test the association between a telomeric cfDNA level and breast cancer risk factors (BRCA1&2 mutations, BMI). Cohen’s *d* was used to calculate the effect size for paired two-sample *t* test using WebPower (http://webpower.psychstat.org) [24]. Linear regression analysis was used to examine the correlation between the plasma telomeric cfDNA level and leukocyte telomere length, as well as between the 10-year risk and either the plasma telomeric cfDNA level or leukocyte telomere length. R squared (*r*^2^) was used for assessing the fit of the regression line. Two-tailed *p* values of less than 0.05 were considered to be statistically significant.

## SUPPLEMENTARY MATERIALS FIGURES AND TABLES



## Abbreviations

cfDNA: cell-free DNA
ctDNA: cell-free tumor DNA
BMI: Body Mass Index
BRCA1&2: BRCA1 and BRCA2
qPCR: quantitative polymerase chain reaction
Ct: threshold cycle
CV: coefficients of variation
LINE: Long INterspersed Element
IBIS: International Breast Intervention Study
