# Supplementary material for: A plasma telomeric cell-free DNA level in unaffected women with BRCA1 or/and BRCA2 mutations: a pilot study
Source: Oncotarget. 2017 Dec 29;9(3):4214–22. doi: 10.18632/oncotarget.23767 (PMC5790533; doi:10.18632/oncotarget.23767)
Supplement: Supplementary file 1 [file oncotarget-09-4214-s001.pdf]

A plasma telomeric cell-free DNA level in unaffected women with BRCA1 or/and BRCA2 mutations: a pilot study

SUPPLEMENTARY MATERIALS

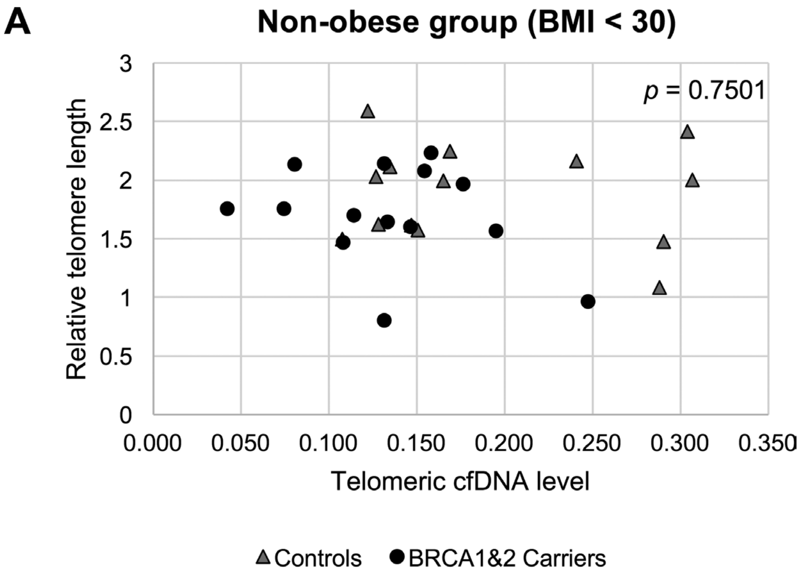

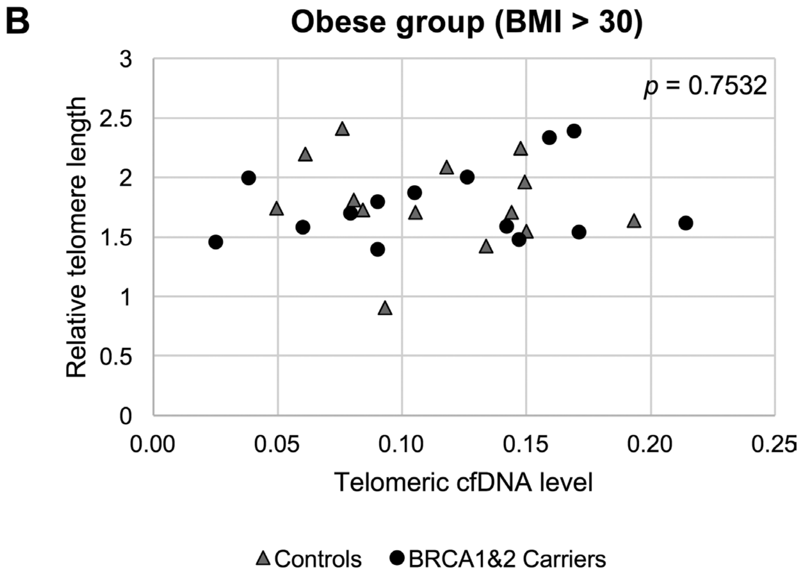

Supplementary Figure 1: There is no significant correlation between a plasma telomeric cfDNA level and leukocyte telomere length. A plasma telomeric cfDNA level was compared with the leukocyte telomere length in the non-obese group (**A**) and the obese group (**B**).

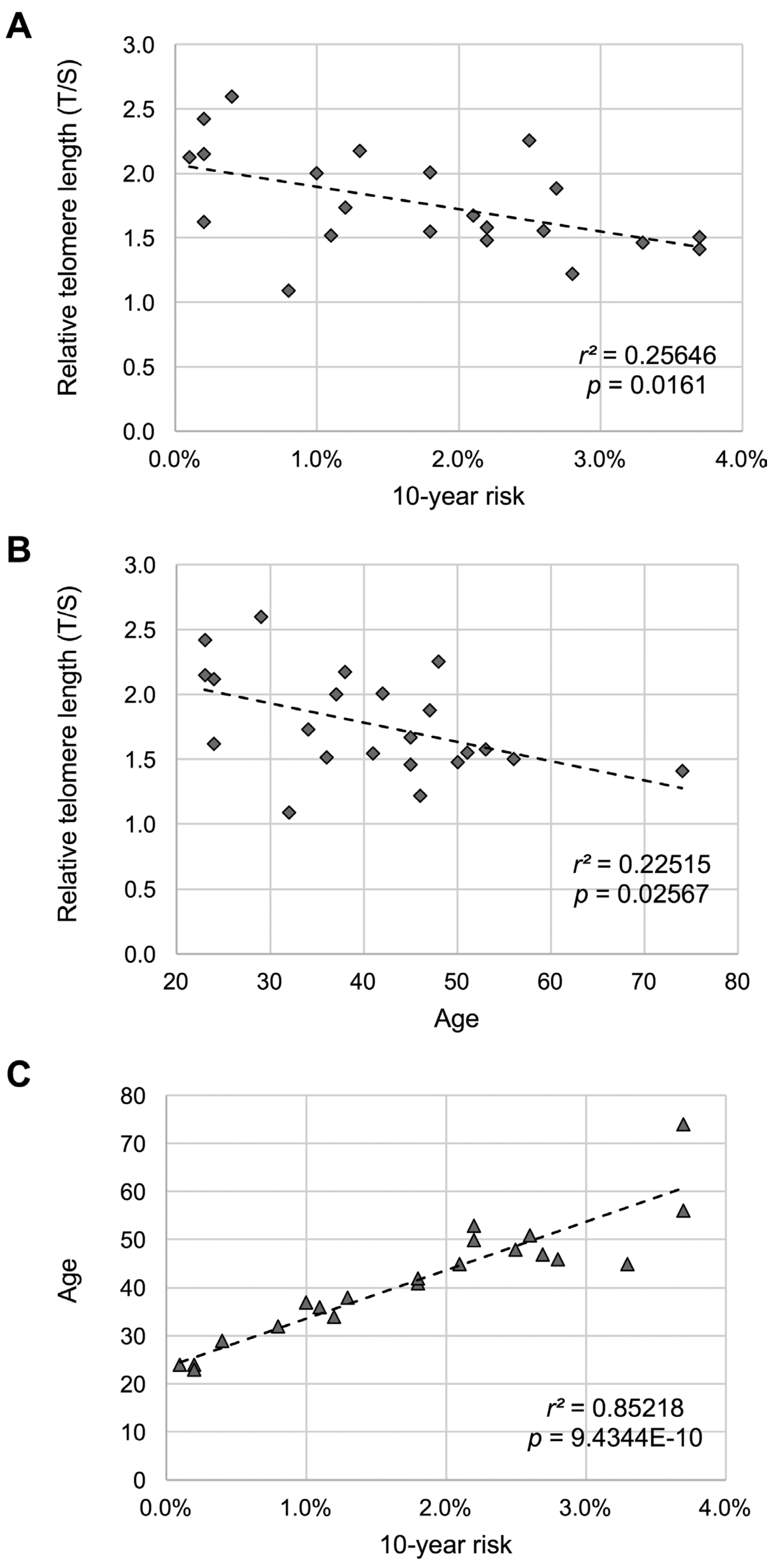

Supplementary Figure 2: Leukocyte telomere length is correlated with the 10-year risk, when the individual 10-year risk is less than 5%. (**A**) Comparison between 10-year risk and leukocyte telomere length. (**B**) Comparison between age and leukocyte telomere length. (**C**) Comparison between 10-year risk and age. Non-obese control women ($n = 22$) were used for the analyses.

Supplementary Table 1: Demographic and clinical characteristics, telomeric cfDNA level, and telomere length of control women

Sample ID	Age	Race	BMI	Current smoking status	Menstrual status	10-year risk	Telomeric cfDNA level (± S.E.*)	Relative telomere length (± S.E.*)
K091-090	23	White	22.5	No	Pre	0.2%	0.304 (± 0.0083)	2.42 (± 0.135)
K103-243	23	White	33.5	No	Pre	4.0%	0.076 (± 0.0049)	2.41 (± 0.114)
K080-224	24	White	21.9	No	Pre	0.2%	0.146 (± 0.0040)	1.62 (± 0.109)
K087-136	24	White	23.3	No	Pre	0.1%	0.134 (± 0.0097)	2.12 (± 0.134)
K095-255	29	White	25.0	No	Pre	0.4%	0.122 (± 0.0025)	2.59 (± 0.126)
K026-379	32	White	18.2	No	Pre	0.8%	0.288 (± 0.0153)	1.09 (± 0.028)
K100-928	34	White	33.3	No	Pre	1.9%	0.105 (± 0.0043)	1.70 (± 0.032)
K096-264	36	White	31.8	No	Pre	1.2%	0.149 (± 0.0080)	1.96 (± 0.169)
K029-318	37	White	20.2	No	Pre	1.0%	0.165 (± 0.165)	2.00 (± 0.200)
K098-843	38	White	34.0	No	Pre	2.6%	0.134 (± 0.0025)	1.42 (± 0.036)
K090-657	38	White	29.8	No	Pre	1.3%	0.240 (± 0.0132)	2.17 (± 0.101)
K106-517	41	White	30.8	No	Pre	3.0%	0.081 (± 0.0037)	1.81 (± 0.032)
K068-569	42	White	22.7	No	Pre	1.8%	0.306 (± 0.0116)	2.00 (± 0.118)
K101-322	45	White	34.4	No	Pre	2.5%	0.049 (± 0.0008)	1.74 (± 0.063)
K107-537	45	White	36.5	No	Post	6.7%	0.093 (± 0.0025)	0.90 (± 0.036)
K003-812	46	White	23.3	No	Pre	2.8%	0.128 (± 0.0023)	1.63 (± 0.110)
K092-428	46	White	32.3	No	Post	2.0%	0.144 (± 0.0077)	1.70 (± 0.146)
K105-353	47	White	35.2	No	Post	9.9%	0.148 (± 0.0073)	2.24 (± 0.061)
K076-708	48	White	24.7	No	Post	2.5%	0.169 (± 0.0033)	2.25 (± 0.061)
K089-090	49	White	31.1	No	Post	2.0%	0.118 (± 0.0079)	2.09 (± 0.053)
K042-373	50	White	21.0	No	Post	2.2%	0.290 (± 0.0119)	1.48 (± 0.039)
K104-297	50	White	36.0	No	Pre	5.1%	0.150 (± 0.0064)	1.55 (± 0.126)
K102-895	53	White	34.5	No	Post	2.5%	0.193 (± 0.0195)	1.64 (± 0.077)
K088-792	56	White	27.4	No	Post	3.7%	0.107 (± 0.0045)	1.50 (± 0.099)
K097-820	62	White	37.1	No	Post	4.9%	0.061 (± 0.0021)	2.20 (± 0.097)
K108-826	65	White	26.6	No	Post	9.5%	0.150 (± 0.0011)	1.57 (± 0.091)
K085-864	70	White	19.5	No	Post	11.0%	0.127 (± 0.0030)	2.03 (± 0.123)
K109-878	74	White	32.8	No	Post	2.9%	0.084 (± 0.0050)	1.73 (± 0.106)

*S.E., standard error of the mean.

Supplementary Table 2: A summary of age, BMI, telomeric cfDNA level, and telomere length in aged-matched control pairs

Pair #	Age	BMI < 30 group			BMI > 30 group		
		BMI	Telomeric cfDNA level	Relative telomere length	BMI	Telomeric cfDNA level	Relative telomere length
1	23	21.1	0.204	2.15	33.47	0.076	2.41
2	24	21.9	0.146	1.62	36.80	0.104	2.15
3	29	25.0	0.122	2.59	32.50	0.148	2.22
4	32	18.2	0.288	1.09	32.95	0.014	1.58
5	34	19.8	0.269	1.73	33.28	0.105	1.70
6	36	22.1	0.214	1.51	31.75	0.149	1.96
7	37	20.2	0.165	2.00	34.01	0.134	1.49
8	41	21.6	0.325	1.55	30.78	0.081	1.81
9	45	20.4	0.329	1.46	34.36	0.049	1.74
10	45	21.4	0.335	1.67	36.49	0.093	0.90
11	46	23.3	0.128	1.22	32.28	0.144	1.70
12	47	24.0	0.195	1.88	35.18	0.148	2.24
13	48	24.7	0.169	2.25	31.09	0.118	2.09
14	50	21.0	0.290	1.48	33.20	0.028	1.76
15	50	24.5	0.127	1.55	36.04	0.150	1.55
16	53	23.7	0.394	1.57	34.54	0.193	1.64
17	61	27.5	0.137	1.41	37.12	0.061	2.20
18	65	26.6	0.150	1.56	33.30	0.117	1.93
19	74	25.2	0.142	1.41	32.81	0.084	1.73
Mean (± S.D.*)	44.2 (± 13.1)	22.7 (± 2.4)	0.217 (± 0.084)	1.67 (± 0.35)	33.80 (± 1.8)	0.105 (± 0.046)	1.83 (± 0.34)

*S.D., standard deviation.

Supplementary Table 3: PCR primer information

Name	Sequences (5′ to 3′)	Concentration (nM)
GC-Telc	CGG CGG CGG GCG GCG CGG GCT GGG CGG TGT TAG GTA TCC CTA TCC CTA TCC CTA TCC CTA TCC CTA ACA	170
GC-Telg	GCC CGG CCC GCC GCG CCC GTC CCG CCG ACA CTA AGG TTT GGG TTT GGG TTT GGG TTT GGG TTA GTG T	170
LINE75_F	CTA TGC AGC CAT AAA AAT GAT GAG TTC ATA TCC	350
LINE75_R	CTG AGA ATG ATG GTT TCC AAT TTC ATC CAT GTC	350
